# Use of a Self-Delivering Anti-CCL3 FANA Oligonucleotide as an Innovative Approach to Target Inflammation after Spinal Cord Injury

**DOI:** 10.1523/ENEURO.0338-20.2021

**Published:** 2021-03-10

**Authors:** Nicolas Pelisch, Jose Rosas Almanza, Kyle E. Stehlik, Brandy V. Aperi, Antje Kroner

**Affiliations:** 1 Department of Neurosurgery, Medical College of Wisconsin, Milwaukee, WI 53226; 2 Department of Microbiology and Immunology, Medical College of Wisconsin, Milwaukee, WI 53226; 3 Clement J. Zablocki Veterans Affairs Medical Center, Milwaukee, WI 53295

**Keywords:** CCL3, FANA ASO, inflammation, novel RNA inhibitor, secondary damage, spinal cord injury

## Abstract

Secondary damage after spinal cord injury (SCI) occurs because of a sequence of events after the initial injury, including exacerbated inflammation that contributes to increased lesion size and poor locomotor recovery. Thus, mitigating secondary damage is critical to preserve neural tissue and improve neurologic outcome. In this work, we examined the therapeutic potential of a novel antisense oligonucleotide (ASO) with special chemical modifications [2′-deoxy-2-fluoro-D-arabinonucleic acid (FANA) ASO] for specifically inhibiting an inflammatory molecule in the injured spinal cord. The chemokine CCL3 plays a complex role in the activation and attraction of immune cells and is upregulated in the injured tissue after SCI. We used specific FANA ASO to inhibit CCL3 in a contusive mouse model of murine SCI. Our results show that self-delivering FANA ASO molecules targeting the chemokine CCL3 penetrate the spinal cord lesion site and suppress the expression of CCL3 transcripts. Furthermore, they reduce other proinflammatory cytokines such as tumor necrosis factor (TNF) and interleukin (IL)-1β after SCI. In summary, we demonstrate for the first time the potential of FANA ASO molecules to penetrate the spinal cord lesion site to specifically inhibit CCL3, reducing proinflammatory cytokines and improve functional recovery after SCI. This novel approach may be used in new treatment strategies for SCI and other pathologic conditions of the CNS.

## Significance Statement

Preserving white matter tissue following spinal cord injury (SCI) is an important therapeutic goal to minimize tissue damage and improve neurologic outcome. Unresolved inflammation contributes to secondary tissue damage. Specifically targeting proinflammatory molecules, such as the chemokine CCL3, in the injured spinal cord can present technical challenges including tissue penetration, stability and efficacy of suppression. Here, we present the use of novel 2′-deoxy-2-fluoro-D-arabinonucleic acid (FANA) antisense oligonucleotide (ASO) molecules as a feasible approach to specifically target proinflammatory molecules in the injured spinal cord.

## Introduction

Spinal cord injury (SCI) causes severe clinical and socioeconomic problems in affected individuals who often face a multifaceted spectrum of complex long-term sequelae, including loss of motor and sensory function and impairment of autonomic nervous system function (Singh et al., 2014). Despite considerable progress in primary and rehabilitative care, curative therapies remain elusive, resulting in SCI remaining a significant cause of disability and mortality (Sekhon and Fehlings, 2001). Tissue damage after SCI is first categorized as primary damage caused by the initial trauma that triggers subsequent pathophysiological cascades and cellular changes classified as secondary tissue damage. Excessive and unresolved inflammation after SCI can contribute to the secondary tissue damage and thus enhance functional impairment. However, the inflammatory response to injury is essential for the clearance of cellular debris and tissue remodeling (Blight, 1994; Donnelly and Popovich, 2008). Factors regulating this inflammatory response after SCI, like the chemokine-ligand/receptor-system, are of particular interest. CCL3, also known as macrophage inflammatory protein (MIP)-1α, is a member of the C-C-subfamily of low molecular weight chemokines. This chemokine is transcriptionally regulated during inflammation (Banisor et al., 2005). It is detectable in most mature hematopoietic cells including monocytes, macrophages (Standiford et al., 1993) and neutrophils (Kasama et al., 1993), as well as microglia and astrocytes (Peterson et al., 1997; Sun et al., 1997). CCL3 is known to play a complex role in the activation and recruitment of immune cells and has been reported to be upregulated after SCI (Bartholdi and Schwab, 1997; Lee et al., 2000; Francos-Quijorna et al., 2017; Zhang and Yang, 2017). Using *CCL3^−/−^* mice, we have previously shown that the absence of CCL3 results in reduced lesion volume and improved functional recovery. Furthermore, we demonstrated a marked reduction of other proinflammatory cytokines like tumor necrosis factor (TNF) and interleukin (IL)-1β (Pelisch et al., 2020). Based on these findings, we concluded that CCL3 may be a promising target for reducing the inflammatory response and secondary damage after SCI. The goal of this project was to specifically target CCL3 in a therapeutically feasible way.

Modulating inflammation inside the injured spinal cord can be challenging, specifically targeting single molecules. Specific inactivation of mRNA can be a valuable approach, but some technical difficulties pose serious challenges. Antisense oligonucleotides (ASOs) are single-stranded, synthetic nucleic acids that recognize and specifically bind mRNA via Watson-Crick base pairing (Bennett and Swayze, 2010). Binding results in degradation of target mRNA by RNase H-dependent mechanisms or prevention of translation or splicing. Although ASO’s potential was established decades ago, actual development of ASO-based drugs faced major challenges (Bhagavati, 2016; Chi et al., 2017; Gustincich et al., 2017). The therapeutic application of ASOs was found to be limited by their high susceptibility to degradation by endogenous nucleases and poor bioavailability. Additionally, ASOs may struggle to cross vascular endothelium, dense extracellular matrix and cell and nuclear membranes to reach their intracellular targets. Thus, introduction of chemical modifications that improve these limitations has become an essential feature in designing ASOs. A variety of sugar modifications contribute to resistance against nucleases and improve binding affinity. 2′-deoxy-2-fluoro-D-arabinonucleic acid (FANA) is an oligonucleotide analog comprised of 2′-deoxy-2′-fluoroarabinonucleotides, which, unlike many 2′-modified analogs that show RNA-like properties, is considered a DNA mimic (Martín-Pintado et al., 2012), forming FANA:RNA hybrids that mirror the structure of the native DNA:RNA hybrid (Denisov et al., 2001). As a result, FANA, and particularly chimeric FANA-DNA ASOs, are able to elicit RNase H-mediated cleavage of target RNA (Lok et al., 2002; Min et al., 2002). Another unique property of FANA ASO molecules is their capability of gymnotic delivery *in vitro* and *in vivo*. Smaldone et al. (2019) demonstrated a ∼99% transfection efficiency using FANA ASO technology in a leukemia cell line .

FANA ASO molecules targeting a variety of genes have been used in various models. For instance, FANA ASOs targeting the HIV-1 genome successfully suppressed viral replication in CD4^+^ T-cells in culture (Takahashi et al., 2019). In other work, FANA ASO molecules were used to confirm a direct link between Abi-1 loss and cell cycle activity in hematopoietic stem cells. Abi-1 protein levels in healthy CD34^+^ cells that were incubated with FANA-*ABI1-*ASO were downregulated >50% compared with scramble control (Chorzalska et al., 2018). *In vivo*, FANA ASO mediated inhibition of phosphodiesterase-4 and phosphodiesterase-7 reduced smoke-induced lung inflammation in mice when administered intratracheally (Fortin et al., 2009). Although the efficacy of ASO in degenerative brain disorders such as Huntington’s disease has been demonstrated (Kordasiewicz et al., 2012; Østergaard et al., 2013), the therapeutic potential of FANA ASOs has never been evaluated for SCI. The present study, to our knowledge, is the first to evaluate anti-inflammatory efficacy of FANA ASOs in the murine spinal cord, using a mouse SCI model of thoracic contusion SCI. Here, we demonstrate the potential of FANA ASO molecules targeting CCL3 to penetrate the spinal cord lesion site, suppress the expression levels of CCL3, reduce levels of proinflammatory cytokines and improve functional recovery after SCI. This novel approach may result in new treatment strategies not only for SCI but also other pathologic conditions of the CNS.

## Materials and Methods

### FANA ASO and administration

Four different FANA ASOs targeting CCL3 used in this study were designed and synthesized by AUM BioTech, LLC based on the CCL3 mRNA sequence obtained from NCBI (NM_011337.2) as follows: CCL3#1: AATAGTCAACGATGAATTGGC; CCL3#2: GACTCTCAGGCATTCAGTTCC; CCL3#3: TCAAGTGAAGAGTCCCTCGAT; CCL3#4: CTACCTAGAATAGCTGTCACC. Scrambled FANA ASO was used as a negative control. Where indicated, FANA ASO with a Cy3 (*in vitro* and histology) or FAM (flow cytometry) fluorescent tags were used.

### Spinal cord contusion injury and FANA ASO treatment

All procedures were approved by and performed in accordance with the Institutional Care and Use Committees of the Clement J. Zablocki Veterans Affairs Medical Center and the Medical College of Wisconsin. Young adult 8- to 10-week-old female wild-type C57BL/6 mice (18–22 g) were obtained from Charles River Laboratories and used for all experiments. For SCI, mice were deeply anesthetized with isoflurane (4% induction, 2.5% maintenance), and a moderate contusion was induced at the T11 thoracic level using the Infinite Horizon Impactor device (Precision Systems and Instrumentation, LLC) with a contusion force of 40 kdyn. Control animals were subjected to a laminectomy without contusion. Animals received subcutaneous carprofen (5 mg/kg) twice daily for 3 d postinjury. Bladders were expressed twice daily until micturition reflexes returned.

Intrathecal injection was performed as previously described (Njoo et al., 2014) at different time points indicated for the individual experiments. Briefly, mice were anesthetized with isoflurane as described above and a lumbar puncture was performed between the groove of lumbar vertebrae L5 and L6 to deliver anti-CCL3 FANA ASO or a scrambled control using a 30-G needle. A tail flick was used as a confirmation that the needle entered the intradural space and 10 μl of solution (10 mg/kg) was slowly administered. The treatment paradigm consisted of one to three doses, with the first dose given immediately after the injury and additional doses every 24 h, where indicated. In one cohort, pretreatment was performed 24 h before injury, followed by another dose immediately after injury and the last dose 24 h later.

### Locomotor assessment

The Basso mouse scale (BMS) open-field locomotor test with a score range of 0–9 (Basso et al., 2006) was used to assess functional recovery during a period of 28 to 35 d. Mice (*n* = 10–13 per group) were evaluated for locomotor function at days 1, 3, 7, 10, and 14 postinjury and weekly thereafter by two individuals blinded to experimental conditions and trained in BMS analysis by Basso’s laboratory at Ohio State University. Consensus scores for each animal were averaged at each time point for a maximum of nine points for the BMS score and 11 points for the sub score, which assesses finer aspects of locomotion. This locomotor assessment was performed in two separate cohorts of mice.

### Tissue collection

For tissue collection, female wild-type mice were euthanized with an overdose of phenytoin/pentobarbital (120 mg/kg) at 1, 3, 7 d after injury for mRNA analysis (*n* = 5–6 mice per time point). After transcardial perfusion with ice-cold, fresh 1× PBS, a 5-mm piece of spinal cord centering on the lesion was dissected out, and immediately snap frozen. RNA was extracted using the RNeasy Lipid Tissue Mini kit (QIAGEN) according to the manufacturer’s instructions, followed by RNA quantification and characterization of purity using Nanodrop 2000 spectrophotometer (Thermo Scientific) and reverse transcribed for 1 μg of RNA using QuantiTect Reverse Transcription kit (QIAGEN).

### Bone marrow-derived macrophages (BMDMs)

BMDMs were generated as previously described (Longbrake et al., 2007) from adult female C57/BL6 mice. Mice were euthanized and femurs removed. Bone marrow was flushed out, homogenized and red blood cells were hypotonically lysed. After washing, cells were cultured in RPMI media containing 20% fetal bovine serum (FBS), 1% penicillin/streptomycin, 1% vitamins (all from Invitrogen), and 10% L-cell-conditioned media (LCCM) as a source of M-CSF for 7 d until cells matured into adherent macrophages. BMDMs were harvested with 0.25% trypsin, re-plated at 1 × 10^6^ cells/well and cultures were stimulated with lipopolysaccharide (LPS; 100 ng/ml, Sigma, L2654) as previously described (Kroner et al., 2014), followed by 10 μmol FANA ASO or scrambled control treatment for 24 h. After stimulation, cells were lysed in RLT buffer (QIAGEN) and mRNA was extracted using the RNeasy Mini kit (QIAGEN) followed by RNA quantification and reverse transcription as described above.

### Q-PCR

Q-PCR was performed in duplicates using a PCR thermal cycler (LightCycler 480 System, Roche) with specific primers using LightCycler 480 Mastermix (Roche). Gene expression levels were analyzed using the ΔΔct method normalized to peptidylprolyl isomerase A (PPIA) as a housekeeping gene and laminectomy as a baseline. Primer sequences for the genes analyzed are as follows: PPIA: ATG TGC CAG GGT GGT GAC TTT A (forward primer 5′–3′); TGT GTT TGG TCC AGC ATT TGC C (reverse primer 5′–3′), CCL3: CAG CTT ATA GGA GAT GGA GCT ATG (forward primer 5′–3′); TCA CTG ACC TGG AAC TGA ATG (reverse primer 5′–3′), TNF: TTG CTC TGT GAA GGG AAT GG (forward primer 5′–3′); GGC TCT GAG GAG TAG ACA ATA AAG (reverse primer 5′–3′), IL-1β: ATG GGC AAC CAC TTA CCT ATT T (forward primer 5′–3′); GTT CTA GAG AGT GCT GCC TAA TG (reverse primer 5′–3′).

### ELISA

Tissue was harvested as described above at days 1, 3, and 7 after SCI (*n* = 5 per time point) and snap frozen. Later, tissue was homogenized as previously described (Sato et al., 2012), and CCL3 and TNF protein concentration was quantified in duplicates using the ELISA kit (CCL3: MMA00, R&D Systems; TNF: Invitrogen, #88-7324).

### Flow cytometry

Flow cytometry was performed 7 d after contusion injury and intrathecal injection of FAM fluorescently labeled FANA ASO molecules as described above to detect the uptake of FANA ASO molecules. Naïve, uninjured mice served as controls. In an additional experiment, flow cytometry was done 1 d after SCI to detect the number of neutrophils in the injured tissue after intrathecal injection of unlabeled CCL3-FANA ASO or scrambled control molecules. Mice were transcardially perfused with PBS, and a 5-mm section of the spinal cord containing the lesion site was dissected Cells were extracted using the Adult Brain Dissociation kit (Miltenyi, #130-107-677) according to the manufacturer’s instructions. Flow cytometry staining was performed following standard protocols as previously described (Kroner et al., 2014). Nonspecific antibody binding was blocked by incubation with 5% bovine serum albumin (BSA), followed by staining with antibodies against CD11b (1:500, 17-4031-82, clone M1/70, rat anti-mouse, APC, Thermo Fisher Scientific) and CD45 (1:500, 25-0451-82, clone 30-F11, rat anti-mouse, PE-Cy7, Thermo Fisher Scientific). Cells from animals at 1 d after SCI without fluorescent FANA ASO injection were additionally stained with anti-Ly6G/C (Gr-1; 1:50, 11-5931-82, Clone RB6-8C5, rat anti mouse, FITC, Invitrogen). Compensation was performed using a compensation particle set (BD Biosciences, #552844). Cells were analyzed using a BD FACS Aria Fusion flow cytometer with FACS Diva software. Results were analyzed using FlowJo software.

### Histology and immunofluorescence

At 5, 7, or 28 d after contusion injury and intrathecal injection with FANA ASO molecules as described above (Cy3 labeled for day 7, unlabeled for days 5 and 28), mice were euthanized as described above, and transcardially perfused with 1× PBS, followed by perfusion fixation with cold 4% paraformaldehyde (PFA); 1-cm-long sections of the spinal cord centering on the lesion site were dissected and postfixed in 4% PFA for 2 h, and cryoprotected in 30% sucrose solution. The cords were then embedded in OCT compound (Sakura Finetek) and serial cross sections (14 μm) were cut and picked up on glass slides (Superfrost Plus Gold; Fisher Scientific). Immunofluorescence labeling was performed as follows: nonspecific binding was blocked by 3 h of incubation in 2% BSA, 0.1% Triton X-100 and 5% normal goat serum. Sections were then incubated overnight at 4°C with the following antibodies: glial fibrillary acidic protein (GFAP) for astrocytes (1:800, Z0334, rabbit anti-mouse; DAKO), CD11b (1:200, MCA711G, rat anti-mouse, Bio-Rad), Neurofilament SMI-32P (1:1500, 801702, mouse anti-rabbit; BioLegend), and NeuN (1:100, MAB377, mouse anti-rabbit, Millipore Sigma). For primary antibodies produced in mouse, 1% of fragment goat anti-mouse IgG (1:22, 115-007-003, Jackson ImmunoResearch) was added to the blocking solution. FluoroMyelin Red for myelin content (1:150; F34652 Thermo Fisher Scientific) was co-stained with GFAP antibody as previously described (Brennan et al., 2016). Slides were then incubated with secondary antibodies (1:400, Alexa Fluor 488 goat-anti-mouse IgG, A11029 and Alexa Fluor 647 goat anti-rabbit; A21245, both Thermo Fisher Scientific) for 1 h at room temperature, followed by washing steps and mounting with DAPI (Vector Labs #6149856).

### Image analysis and quantification

Images were captured using a Nikon Eclipse E600 equipped with a digital camera or a Leica TCS SP8 Laser Scanning Confocal Microscope. Analyses of histologic sections were performed using ImageJ (ImageJ-win64 2.0). The epicenter of injury in all spinal cords was identified, followed by quantification of myelin loss using Fluoromyelin-stained cross sections of the spinal cord. Areas devoid of GFAP labeling were measured at 112-μm intervals by calculating the GFAP-devoid area as a percentage of the total area of the cross section.

For the quantification of astrocytes, macrophages/microglia and SMI-32+ profiles, a region of interest covering a 290 × 290 μm area was captured laterally perilesional to the lesion core at the epicenter site with 14 z-stack sections (1 μm) spanning the complete axial thickness (14 μm).

DAPI nuclei of astrocytes, microglia/macrophages were manually quantified through a continuous viewing of all z-stack sections. This allowed for greater resolution and ability to distinguish individual cells in presence of complex ramified cellular morphologies following damage progression. SMI-32+ endbulbs were quantified following a similar approach. All analyses were done by a blinded investigator.

### Statistical analyses

Comparisons between two datasets were analyzed by Student’s *t* test. Data with more than two variables were analyzed by ordinary one-way ANOVA (different time points in different samples) two-way repeated measures ANOVA (different time points in the same samples) with *post hoc* Tukey’s or Dunnett’s analysis. χ^2^ test was used for testing relationships categories (GraphPad Prism version 8). All *p*s < 0.05 were considered statistically significant.

We also applied estimation based on confidence intervals (CIs). For this, we directly uploaded raw data in https://www.estimationstats.com/ and included the results (Ho et al., 2019). The mean difference for two comparisons is reported. Five thousand bootstrap samples were taken; the CI was bias-corrected and accelerated. To measure the effect size, we included unbiased Cohen’s *d* (standardized mean difference).

## Results

### FANA ASO molecules targeting CCL3 penetrated BMDMs in a gymnotic fashion and reduced CCL3 expression *in vitro*

**Figure 1.**
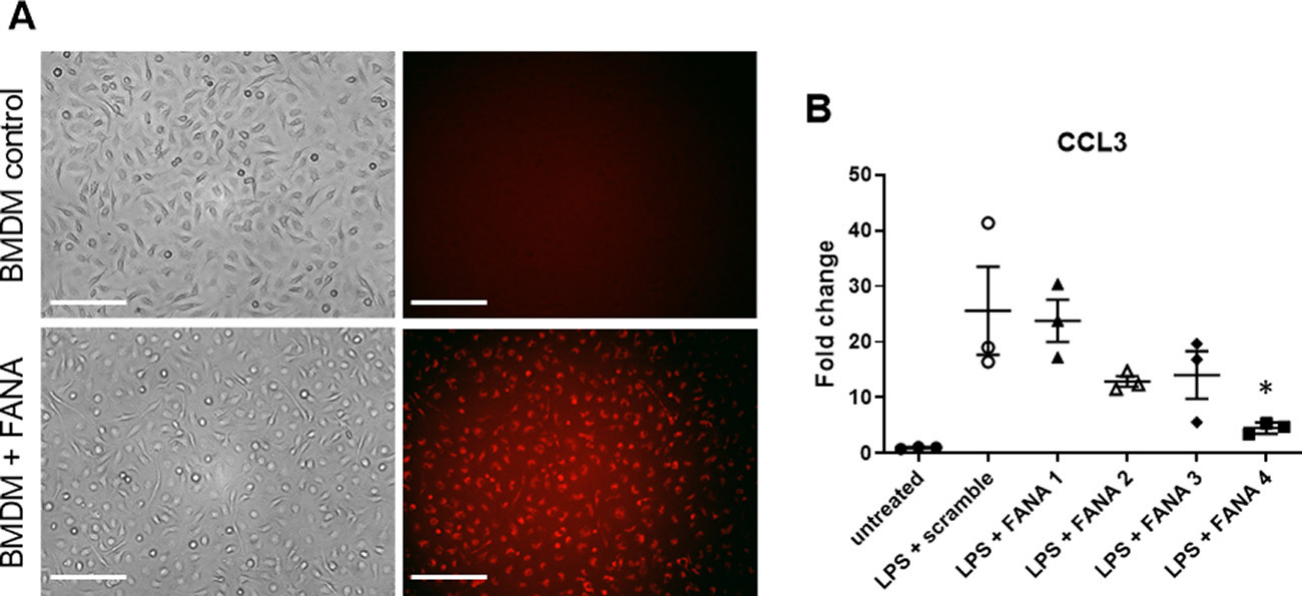
FANA ASO molecules penetrate BMDMs in a gymnotic fashion and reduce CCL3 expression *in vitro*. ***A***, When added to the tissue culture media, fluorescently labeled FANA ASO molecules penetrate BMDM cells after 2 h of incubation (red fluorescence, lower panel). The upper panel demonstrates the absence of fluorescence when no FANA ASO molecules were added to the media. Scale bar: 50 μm. ***B***, Assessment of CCL3 knock-down in LPS stimulated BMDMs using four different FANA ASOs molecules. CCL3 mRNA expression levels were compared between LPS-treated BMDMs in the presence of scrambled control FANA ASO or the individual anti-CCL3 FANA ASOs. CCL3 expression levels were unchanged in the presence of FANA-CCL3#1, FANA-CCL3#2, and FANA-CCL3#3. Treatment with FANA-CCL3#4 resulted in a significant reduction of CCL3 expression by ∼80%. Untreated BMDMs are included for reference; *n* = 3/group; *p < 0.05. Error bars are represented as SEM.

To our knowledge, this is the first study using CCL3 targeting FANA ASO molecules. We first investigated whether fluorescently labeled FANA ASO molecules were able to penetrate BMDMs in a gymnotic fashion. For this, FANA ASO molecules were added directly to the cell culture media at a final concentration of 10 μm. After 2 h of incubation, all cells showed red fluorescence, indicating FANA ASOs had penetrated them compared with media without FANA ASOs (Fig. 1*A*). Next, we assessed the potential of CCL3 knock-down in LPS stimulated BMDMs using four different FANA ASO molecules. BMDMs were stimulated with LPS to increase CCL3 expression, which is very low in unstimulated cells. CCL3 mRNA expression levels were compared between LPS-treated BMDMs in the presence of scrambled control FANA ASOs and the individual anti-CCL3 FANA ASO. CCL3 expression levels were not significantly changed in the presence of FANA-CCL3#1, FANA-CCL3#2, and FANA-CCL3#3. However, treatment with FANA-CCL3#4 resulted in a significant reduction of CCL3 expression by ∼80% (Fig. 1*B*). Accordingly, all following inhibition experiments were performed using FANA-CCL3#4.

### Fluorescently labeled FANA ASOs could be detected at the SCI lesion site after intrathecal injection

**Figure 2.**
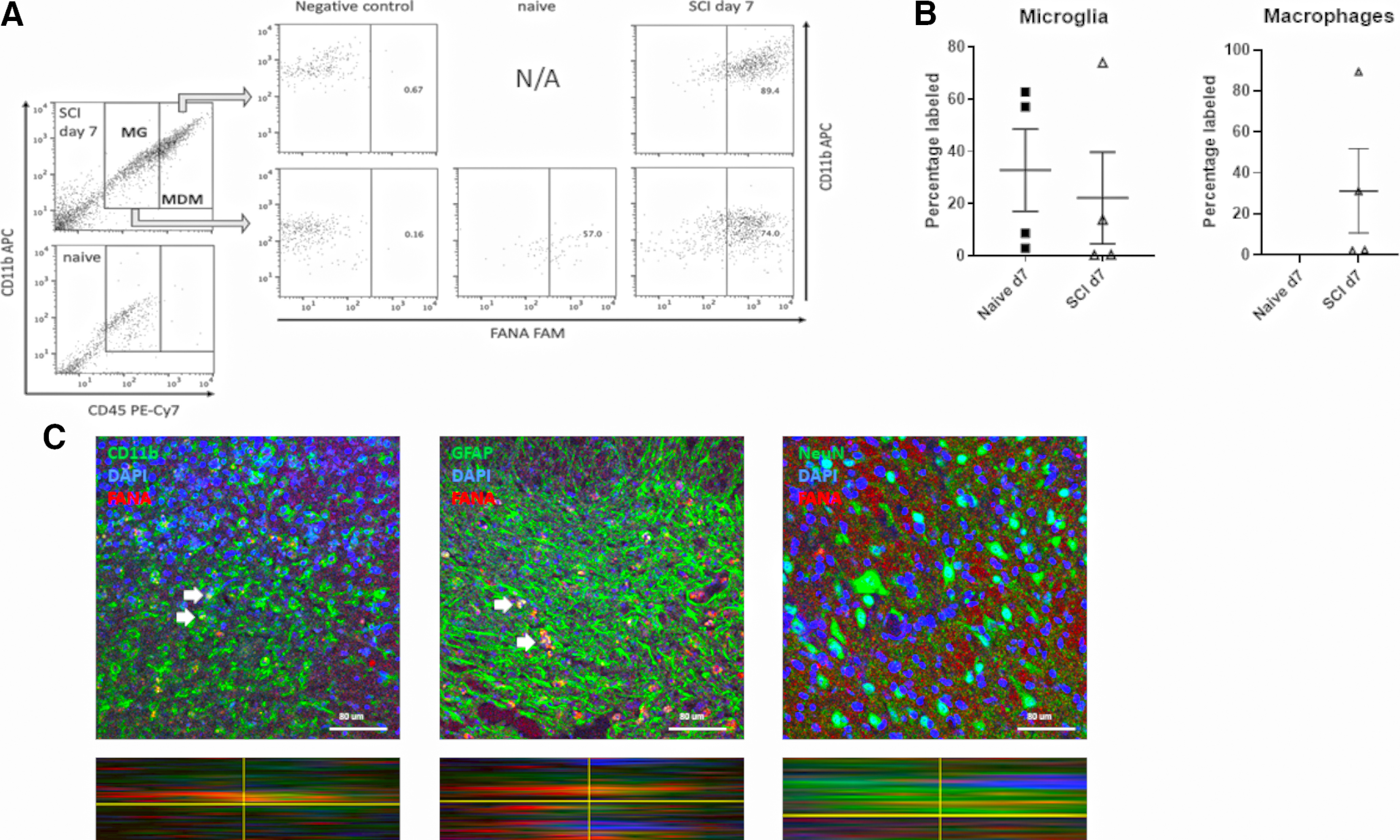
Fluorescently labeled FANA ASOs are present at the SCI lesion site after intrathecal injection. ***A***, Representative images of flow cytometry on cells from a section of spinal cord tissue centering on the lesion or spinal cord tissue from naive mice. Green fluorescence derived from FAM-labeled FANA was detectable in gated cell populations for CD11b+/CD45low microglia (MG) and CD11b+/CD45high MDMs. MDM are not detected in naive tissue. ***B***, Quantification of percentages of MG and MDM containing FAM-labeled FANA; *n* = 4/group, error bars are represented as SEM. ***C***, Seven days after SCI and intrathecal injection, labeled FANA ASO was detectable at the lesion site (red staining). Confocal images with orthogonal view demonstrate uptake of FANA molecules in a non- cell specific manner, as shown by the presence in CD11b+ macrophages/microglia and GFAP+ astrocytes. No uptake was detected in NeuN+ neurons; *n* = 4–5/group. Scale bars: 80 μm.

Next, we assessed the feasibility of intrathecal delivery of FANA ASO molecules. The rationale for intrathecal administration was to reduce systemic exposure and reduce anti-inflammatory effects in the periphery. To assess whether FANA ASOs were reaching the lesion site, we injected 10 mg/kg of fluorescently labeled FANA ASOs intrathecally in SCI and naive, uninjured mice. Seven days after injection, we verified the presence of FANA ASO at the lesion site (Fig. 2). Using flow cytometry, we could demonstrate the presence of FANA ASO molecules in CD11b+/CD45low microglia (MG) and CD11b+/CD45high monocyte-derived macrophages (MDMs; Fig. 2*A*). The percentage of labeled cells showed wide variability, possibly indicative of less successful injections.

The percentage of labeled microglia was similar between naive and injured mice while very low MDMs were detected in naive mice (Fig. 2*B*). FANA ASO labeling at the lesion site was also assessed histologically. The presence of red (Cy3)-fluorescent labeling, an indication of FANA ASO uptake, was observed in CD11b+ macrophages/microglia and GFAP+ astrocytes, demonstrating that FANA ASOs are not taken up by any specific cell type. However, no FANA ASO uptake was detected in NeuN+ neurons (Fig. 2*C*).

### The inflammatory response following SCI was reduced after FANA ASO injection

**Figure 3.**
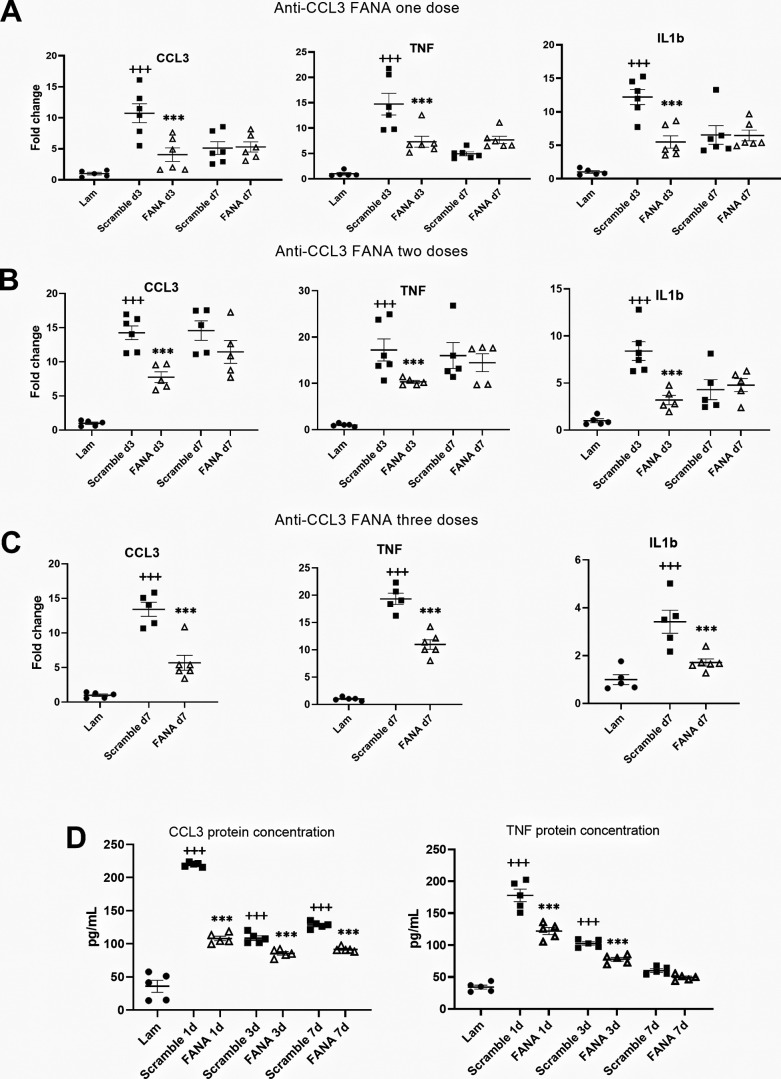
CCL3 expression levels following SCI were reduced after FANA ASO injection. ***A***, A single injection of FANA ASO CCL3#4 resulted in significant suppression of CCL3 mRNA expression in the injured spinal cord compared with scrambled control at day 3 postinjury. This reduction was accompanied by the suppression of TNF and IL-1β expression, indicating a reduction of the proinflammatory tissue reaction. ***B***, When using a two-injection paradigm, CCL3, TNF, and IL-1β mRNA were also suppressed at day 3. ***C***, When three doses were administered, a sustained suppression of CCL3 was detected at day 7, reducing CCL3 levels by ∼60%. Both TNF and IL-1β gene expression levels were reduced by ∼50%. ***D***, Suppression of CCL3 and TNF in the injured spinal cord was also measured the protein level using ELISA at days 1, 3, and 7 and days 1 and 3 after SCI, respectively; *n* = 5/group; +++*p* < 0.001 (compared with laminectomy control); *p < 0.05, ***p < 0.001 (compared with scrambled control of the same time point). Error bars are represented as SEM.

After establishing that FANA ASOs penetrated the lesion site after lumbar intrathecal injection, we assessed the efficacy of suppressing CCL3 expression at different time points after injury. A single injection of FANA ASO CCL3#4 resulted in significant suppression of CCL3 mRNA in the injured spinal cord compared with injection of scrambled control at day 3 postinjury. This reduction was accompanied by the suppression of TNF and IL-1β transcripts, indicating a reduction of the proinflammatory tissue environment. However, this effect was not sustained beyond day 3 (Fig. 3*A*). To achieve a longer lasting suppression of CCL3, we used a two-injection paradigm immediately after surgery and 24 h postinjury, which confirmed CCL3, TNF, and IL-1β suppression only at day 3 (Fig. 3*B*). However, when three injections were administered every 24 h for 3 d, sustained suppression of CCL3 was detected at day 7, reducing CCL3 levels by ∼60%. Both TNF and IL-1β expression levels were also reduced by ∼50% (Fig. 3*C*). Suppression of CCL3 in the injured spinal cord was confirmed at the protein level using ELISA at 1, 3, and 7 d after SCI. TNF protein suppression was sustained at days 1 and 3. At day 7, overall TNF expression had returned to baseline expression levels and was not different between CCL3 FANA ASO and control-treated mice (Fig. 3*D*).

### Treatment with anti-CCL3 FANA ASOs resulted in mild functional improvement

**Figure 4.**
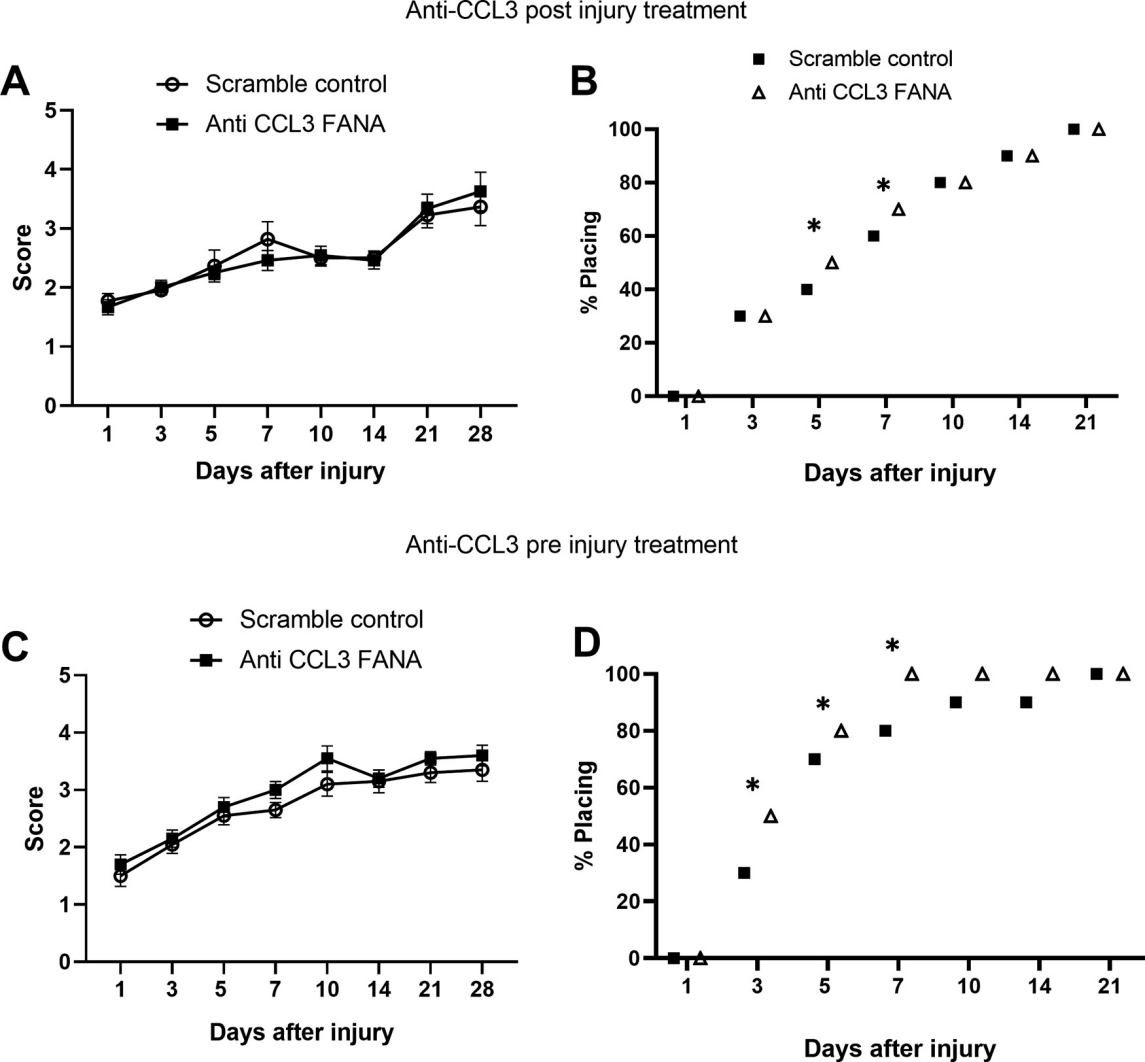
Treatment with anti-CCL3 FANA ASO resulted in mild functional improvement. ***A***, BMS scores did not show significant improvement compared with control animals when anti CCL3-FANA was administered three times starting right after injury. ***B***, However, a significantly higher percentage of CCL3-FANA ASO-treated mice performed plantar placement on days 5–7 after injury, compared with control animals. To ensure sufficient time to knock-down CCL3, we also investigated the efficacy of CCL3-FANA ASO pretreatment, with the first dose given 24 h before injury, followed by two more injection immediately after injury and 24 h later. ***C***, BMS scores were not significantly different in treated versus control animals when anti CCL3-FANA was administered three times starting 24 h before injury. ***D***, The pretreatment group, however, showed a significant increase of plantar placement at days 3 through 7 after injury compared with controls; *n* = 10/group in preinjury treatment paradigm, *n* = 12/group in postinjury treatment. Data points are represented as mean ± SEM. Note that bars in ***B***, ***D*** represent the total percentage of mice from each group capable of plantar placement, thereby not allowing for error bars; *p < 0.05, χ^2^ test.

We then assessed the therapeutic potential of CCL3 inhibition using FANA ASO. To this end, we induced a moderate contusion injury at T11, followed immediately by intrathecal injection of CCL3-FANA ASO or scrambled control, and repeated injections after 24 and 48 h. BMS scores in this cohort did not show significant improvement compared with control animals (Fig. 4*A*). However, a significantly higher percentage of CCL3-FANA ASO-treated mice performed plantar placement on days 5–7 after injury, compared with control animals (Fig. 4*B*). To ensure sufficient time for CCL3 knock-down, we also investigated the efficacy of CCL3-FANA ASO pretreatment, with the first dose given 24 h before injury, followed by two more injections immediately after injury and after 24 h. BMS scores were also not significantly different in treated versus control animals (Fig. 4*C*), but the pretreatment group showed a significant increase of plantar placement at days 3 through 7 after injury compared with controls (Fig. 4*D*), indicating that CCL3 inhibition with FANA ASOs may result in a mild functional improvement.

### Treatment with anti-CCL3 FANA ASOs reduced CD11b+ macrophages at the lesion site

**Figure 5.**
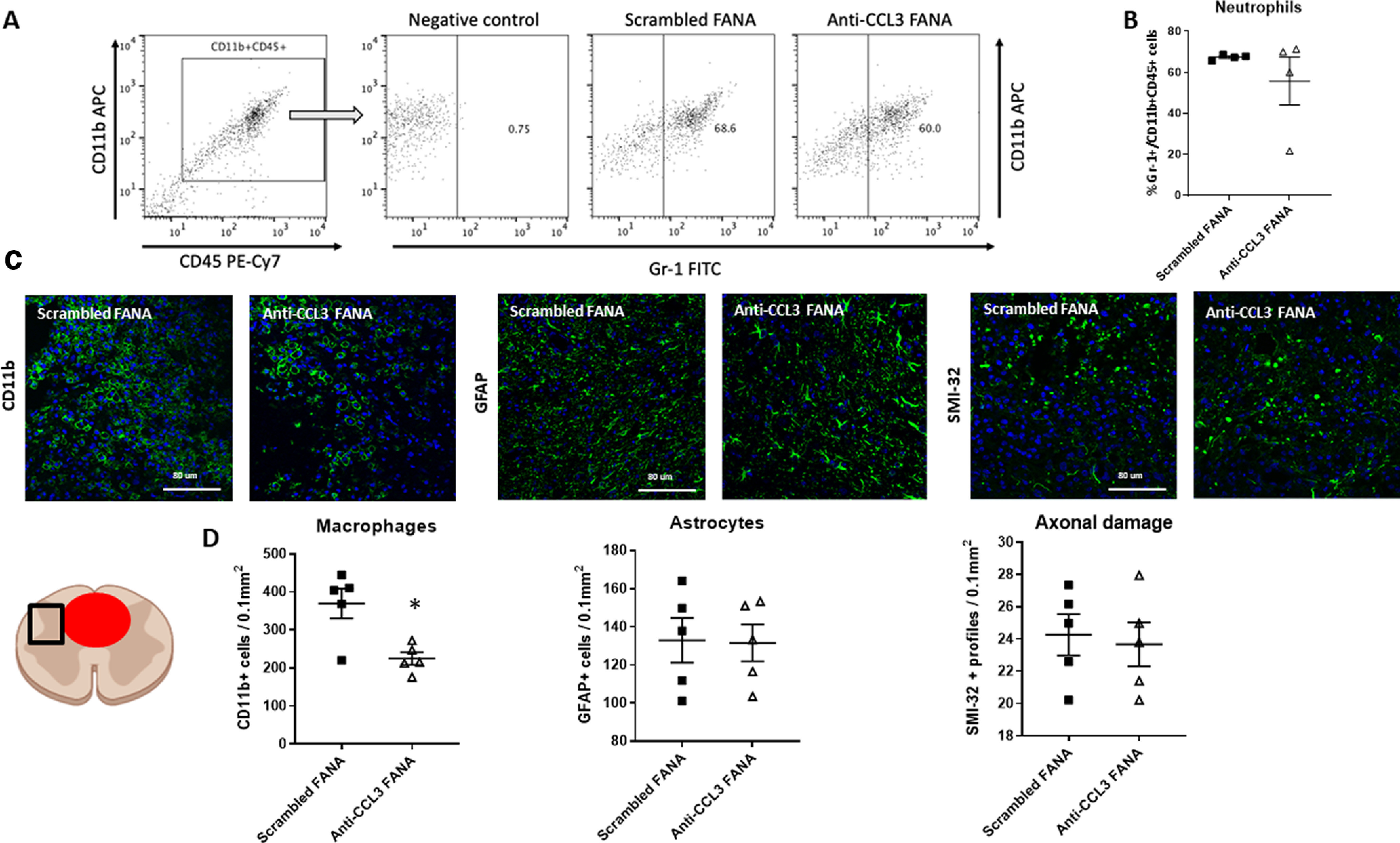
Treatment with anti-CCL3 FANA ASOs reduced CD11b+ macrophages at the lesion site. *A*, B. Flow cytometry 1 d after SCI in anti-CCL3-FANA ASO and control-treated animals did not reveal a significant difference in Gr-1+ neutrophils. Numbers were assessed in the CD11b+CD45+ gated cell population. The negative control was stained with CD11b+CD45+ in absence of the Gr-1 antibody. ***A***, Representative flow cytometry images. ***B***, Quantification of CD11b+CD45+Gr-1+ neutrophils. ***C***, Representative images of CD11b+ macrophages/microglia, GFAP+ astrocytes, and SMI-32+ profiles as indicators of axonal damage adjacent to the lesion epicenter in anti-CCL3-FANA ASO and control-treated animals. The schematic indicates the location of the quantified region (square) next to the lesion (red circle). ***D***, Quantification revealed a significant reduction of CD11b+ macrophages/microglia in the anti-CCL3-FANA ASO treatment group, while neither astrocytes nor indicators of axonal damage were different between groups; *n* = 5/group, data points are represented as mean ± SEM; *p = 0.01. Scale bars: 80 μm.

Next, we assessed whether treatment with CCL3-FANA ASO influenced the cellular composition at the lesion site. Gr-1+ cells were quantified using flow cytometry 1 d after SCI. No difference in neutrophil numbers was detected between the groups (M_diff_ = −11.6 [95.0% CI −36.8, 2.45], *d *=* *0.7, *p *=* *0.43 for *t* test; Fig. 5*A*,*B*). Gr-1 detects both Ly6G+ neutrophils and Ly6C+ monocytes. One day after injury, however, the majority of Gr-1+ cells are likely to be neutrophils.

We then performed histologic assessment of immune related cells and markers of axonal damage at day 5 after SCI. This time point was chosen because of the maintained high inflammatory reactivity of the tissue combined with the presence of macrophages in the tissue as well as signs of axonal degeneration. We quantified CD11b+ macrophages/microglia, GFAP+ astrocytes and SMI-32+ retraction bulblike structures adjacent to the lesion epicenter. Interestingly, the amount of CD11b+ macrophages/microglia was significantly reduced in CCL3-FANA ASO-treated animals, in agreement with the reduced inflammatory response (M_diff_ =−1.45e + 02 [95.0% CI −2e+02, −38.5], *d *=* *2.15, *p *=* *0.01 for *t* test). Astrocyte numbers and indicators of axonal damage (SMI-32+ profiles) were not different between groups (M_diff_ = −1.43 [95.0% CI −27.6, 24.7], *d *=* *0.06, *p *=* *0.94 for *t* test) and (M_diff_ −0.5 [95.0% CI −3.2, 2.4], *d *=* *0.021, *p *=* *0.72 for *t* test; Fig. 5*C*,*D*).

### CCL3 inhibition with specific FANA ASOs did not significantly reduce tissue damage after SCI

**Figure 6.**
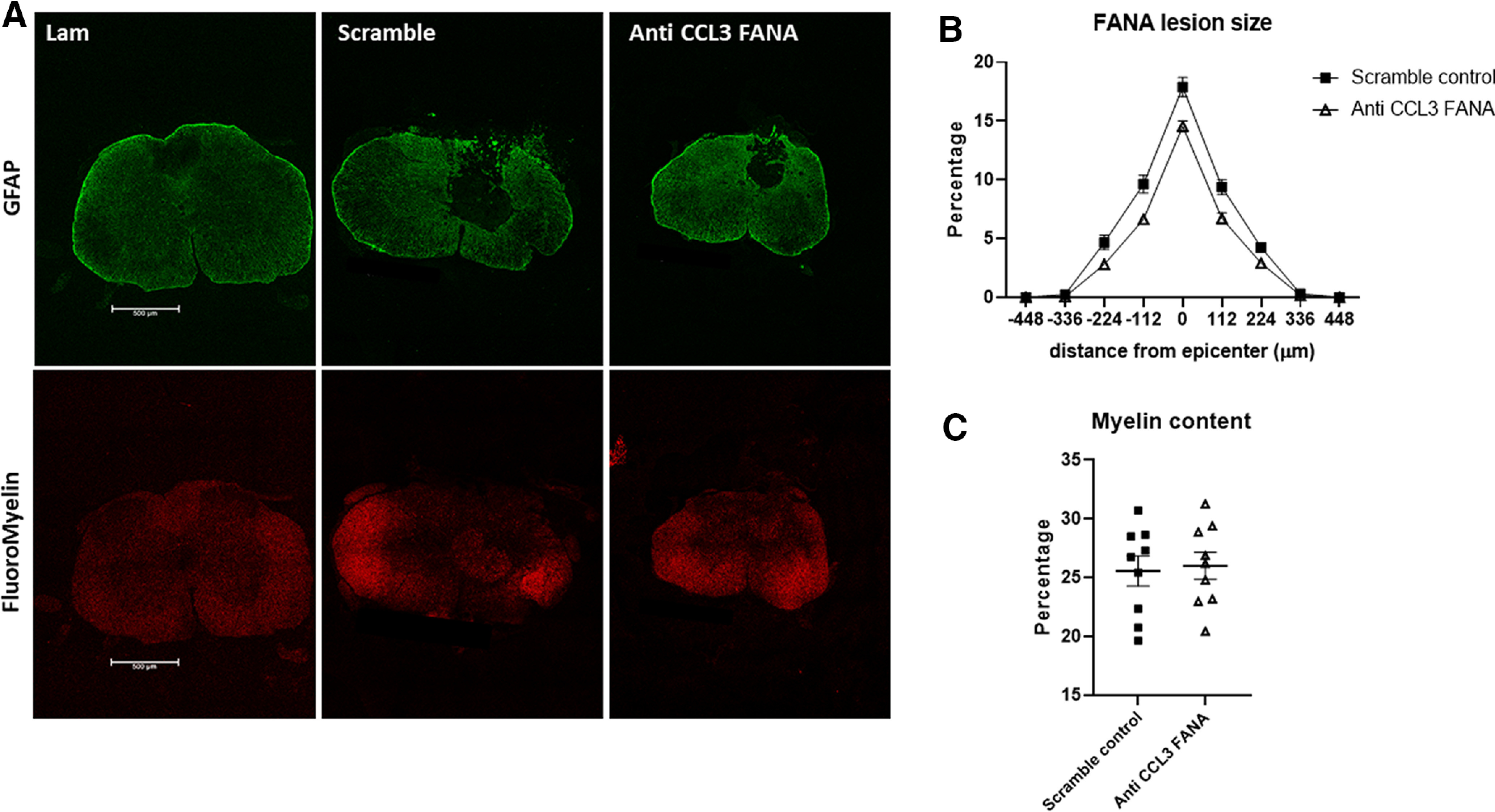
CCL3 inhibition with FANA ASOs did not significantly reduce tissue damage after SCI. Quantification of areas devoid of GFAP immunoreactivity at day 28 after SCI did not show a significant difference in lesion area in anti-CCL3-FANA ASO-pretreated mice compared with control mice. ***A***, Representative images of GFAP and FluoroMyelin stained spinal cord cross sections. ***B***, Quantitative analysis of the lesion volume in CCL3-FANA ASO-pretreated mice and control mice. ***C***, No differences in myelin content, as shown with FluoroMyelin Red staining, were detected between groups; *n* = 9/group. Data points are represented as mean ± SEM. Scale bars: 500 μm.

Finally, we quantified lesion sizes in the injured spinal cords of CCL3-FANA ASO-treated and control mice. Immunofluorescent staining for GFAP and FluoroMyelin was used to examine the lesion size as well as the amount of preserved myelin in the injured spinal cords (Fig. 6*A*). Quantitative analysis of the lesion volume did not show a significant reduction in lesion size in CCL3-FANA ASO-pretreated mice (Fig. 6*B*). No differences in myelin content, as shown with FluoroMyelin Red staining, were detected between groups (Fig. 6*C*).

## Discussion

Uncontrolled inflammation plays a critical role during secondary tissue damage following traumatic SCI. In particular, proinflammatory cytokines and chemokines have been shown to play a main role in exacerbating the secondary damage after the primary injury. Specifically targeting proinflammatory molecules in the injured spinal cord can present technical challenges in regard to tissue penetration, stability and efficacy of suppression. The use of specific FANA ASO molecules might be a feasible approach to circumvent these issues. Here, we set out to determine the efficacy of this innovative strategy to specifically target inflammatory molecules after SCI. Taken together, our experiments demonstrate both the feasibility in using FANA ASO molecules after SCI and their anti-inflammatory potential in the injured tissue.

Our results indicate that intrathecal administration of anti-CCL3 FANA ASO treatment provides a significant downregulation of CCL3 and others proinflammatory cytokines. This is of interest because it demonstrates the local efficacy of CCL3 suppression as well as the impact on the inflammatory tissue environment. Moreover, targeting CCL3 using this technology led to a slight improvement of locomotor recovery. These results support our previous data showing the role of CCL3 in modulating the inflammatory response and secondary degeneration after SCI. This warrants further studies to examine this technology to target CCL3 and other inflammatory molecules for reducing secondary damage and improving functional recovery.

In the past years, ASOs have been validated as therapeutic agents to generate mRNA selective knock-downs both *in vitro* and *in vivo*. However, successful application was limited by critical technical issues. These issues included insufficient stability of RNA inhibitors because of nucleases degradation activity, and poor cellular delivery as a result of low cellular uptake from inefficient crossing of cellular membranes. For this, next generation ASOs, such as FANA ASO molecules, have been developed to improve nuclease resistance, increase binding affinity, and to enhance biostability. One of the most important modification to the biological function of FANA ASO molecules is their ability to penetrate cells without the necessity of delivery vehicles required in transfection approaches. Our results revealed that FANA ASO molecules targeting CCL3 can indeed penetrate BMDMs in gymnotic fashion, as previously described for other cell types *in vitro* (Schmidt et al., 2019; Smaldone et al., 2019; Suwanmanee et al., 2019; Takahashi et al., 2019; Baby et al., 2020; Margiotta and Howard, 2020). We also demonstrated the successful suppression of CCL3 expression in BMDMs by CCL3 specific FANA ASOs. Out of four molecules targeting different areas of the CCL3 mRNA that we investigated, one molecule mediated significantly reduced CCL3 expression, while three others did not significantly suppress CCL3 *in vitro*.

As a next important step, we assessed the feasibility of *in vivo* application of these FANA ASO molecules after SCI and in uninjured animals. We observed gymnotic tissue penetration after intrathecal injection, when fluorescently labeled FANA ASO molecules were detected in the spinal cord, primarily at the lesion site. Using flow cytometry, FANA uptake could be detected in CD11b+CD45low microglia (MG) of naive mice and 7 d postinjury mice. SCI mice also showed labeling of CD11b+CD45high MDMs, a cell population which, as expected, are not present in naive animals. CD11b is a myeloid cell marker commonly used to identify MG and MDM in combination with CD45 expression levels. Both CD11b and CD45 are also expressed on neutrophils. However, at this time point after SCI, the majority of myeloid cells in the tissue are expected to be microglia or MDM.

The percentage of labeled cells showed a high variability within the group, which may be explained by less successful intrathecal injections. Further work will have to be done to optimize administration and availability of FANA ASO molecules in the injured spinal cord.

Fluorescently labeled FANA ASO could also be detected histologically, with the highest concentrations being close to the lesion site. Its uptake was not cell specific, as we observed FANA ASO positive macrophages/microglia and astrocytes. Interestingly, no penetration was detected in NeuN+ neurons, which could be explained by their localization with some distance to the lesion epicenter.

When assessing the efficacy of CCL3 suppression after intrathecal injection, we achieved a gene expression reduction of 50–60% in the injured tissue 3 d after SCI. This suppression level was comparable when one or two doses of FANA ASO were administered intrathecally. However, neither one nor two doses of FANA ASO, immediately and 24 h after injury, were sufficient to significantly suppress CCL3 expression levels persistently for 7 d after SCI. When a third dose was added 48 h following injury, CCL3 expression levels were significantly reduced by 62% in the injured tissue 7 d after injury. This significant reduction of CCL3 after three doses was confirmed on the protein level using ELISA. FANA ASO mediated mRNA suppression was observed to the same extent in a model of Huntington’s disease, where the suppression persisted for 12 weeks after continuous infusion for two weeks (Kordasiewicz et al., 2012). *In vitro,* sustained antisense activity has been described for other FANA ASO molecules for four to 13 d (Ferrari et al., 2006; Takahashi et al., 2019).

Interestingly, the levels of the proinflammatory cytokines TNF and IL-1β closely resembled the levels of CCL3 after different administration paradigms. TNF concentration was confirmed on the protein levels to be reduced at days 1 and 3 after SCI. No significant reduction was detectable at day 7. At this time, overall TNF levels had returned to baseline expression. We could also demonstrate that the treatment with anti-CCL3 FANA ASO reduced the number of macrophages adjacent to the lesion 5 d after injury, a finding that closely resembles our observation in CCL3^−/−^ mice (Pelisch et al., 2020).

These findings support the importance of CCL3 as an inflammatory chemokine, which regulates the production of proinflammatory cytokines and the chemotactic mobilization of immune cells into inflammatory tissues (Baba and Mukaida, 2014). It is important to consider that all experiments were performed in 8- to 10-week-old mice. While this age group is frequently used in SCI research, it has been described that the inflammatory response after SCI in young mice is less pronounced compared with older mice (Zhang et al., 2015). The use of different age groups may reveal different effects on the inflammatory response in future experiments.

After demonstrating the efficacy of FANA ASO molecules to reduce CCL3 expression following SCI *in vivo*, we assessed the impact on functional recovery. Open field locomotor (BMS) score showed no significant differences in mice receiving anti-CCL3 FANA ASO treatment compared with scrambled control. However, a significantly higher percentage of mice treated with CCL3-FANA ASO performed plantar placement on days 5–7 after injury, compared with control animals, confirming that CCL3 knock-down before and/or after injury with CCL3-FANA ASO can result in a mild functional improvement. However, treatment with FANA ASO did not result in a significant reduction of lesion size. Furthermore, no difference in axonal damage, assessed by quantification of SMI-32+ profiles, was detected at day 5 after injury. These results are in line with the absence of preserved behavioral improvement at later time points after injury. The absence of more pronounced behavioral differences or tissue preservation could be explained by the level of CCL3 suppression. Complete absence of CCL3 in CCL3 deficient mice results in a slightly but significantly reduced functional recovery and reduction of lesion sizes (our unpublished data). However, the suppression of CCL3 in our SCI model reached ∼60%, which could lead to a reduced functional impact.

Our previous work in CCL3^−/−^ mice demonstrated improved locomotor recovery and reduced tissue damage. It is therefore tempting to speculate that increased efficacy of suppression would result in more pronounced tissue preservation and functional recovery.

In summary, for the first time, this study demonstrates the feasibility and potential of the use of FANA ASO molecules *in vivo* targeting specific molecules in the injured or pathologically altered spinal cord tissue. The use of this technology could provide important treatment options to mitigate secondary damage following SCI as well other diseases of the brain and spinal cord. Moreover, we demonstrate that targeting CCL3 using a novel mRNA inhibitor, FANA ASO, results in a reduced proinflammatory response followed by a mild but significant functional improvement after SCI.

## Synthesis

Reviewing Editor: Jeffery Twiss, University of South Carolina Decisions are customarily a result of the Reviewing Editor and the peer reviewers coming together and discussing their recommendations until a consensus is reached. When revisions are invited, a fact-based synthesis statement explaining their decision and outlining what is needed to prepare a revision will be listed below. The following reviewer(s) agreed to reveal their identity: Ashley Kalinski, Dylan McCreedy.

The reviewers were overall very positive for this resubmission. In particular, they noted that this is the first use of FANA ASO molecules to regulate neuroinflammation and represents a critical first step in the use of FANA ASOs to target pro-inflammatory cytokines after spinal cord injury. Both reviewers felt that their major concerns were answered, and that the new data provide critical insight into the role of CCL3 in inflammation occurring after spinal cord injury, as well as the efficacy of the described anti-CCL3 FANA therapy. So, with the revisions, both reviewers and the reviewing editor consider that the work adds value to the field of neural repair and brings a novel direction to pursue. However, one reviewer raised minor concerns that I think need to be addressed prior to further consideration. These minor issues are outlined below and would only require editorial review.

1. There was an issue with Figure 2. It appears to have not loaded or converted properly, so the changes in the figure could not be evaluated. Please double check the conversion for all figures on your resubmission. I suggest that you verify that a version saved on your computer reopens correctly. In the event of issues, please contact the eNeuro staff and cc’ me on any queries.

2. CD11b can label more than macrophages and microglia (it can label all myeloid lineage cells including neutrophils) and GR-1 can label more than neutrophils (it also labels Ly6C+ monocytes). This is a minor concern, but it should be addressed in the text how the authors are able to convey specificity to the indicated cell types with these markers or briefly mentioned as a potential limitation in the discussion.

3. For Figure 6B, the denominator for the y-axis title is not clear. I’m assuming it should read "% GR-1+ cells out of CD11b+CD45+ cells"

4. For the images used in Figure 6C, it would be helpful to add schematic to indicate location of the image taken within the section.
